# Comparison of total lipids and fatty acids from liver, heart and abdominal muscle of scalloped (*Sphyrna lewini*) and smooth (*Sphyrna zygaena*) hammerhead sharks

**DOI:** 10.1186/2193-1801-3-521

**Published:** 2014-09-12

**Authors:** Bruce Clement Davidson, Wynand Nel, Afsha Rais, Vahid Namdarizandi, Scott Vizarra, Geremy Cliff

**Affiliations:** Saint James School of Medicine, PO Box 318, Albert Lake Drive, The Quarter, Anguilla, AI-2640 British West Indies; KwaZulu-Natal Sharks Board, and Biomedical Resource Unit, University of KwaZulu-Natal, Private Bag 2, Umhlanga Rocks, 4320, Durban, 4056 KwaZulu-Natal South Africa

**Keywords:** Buoyancy, Fatty acids, Hammerhead shark, Ketones, Lipids, n-3 polyunsaturates, 22:6n-3

## Abstract

Liver, heart and abdominal muscle samples from scalloped (*Sphyrna lewini*) and smooth (*Sphyrna zygaena*) hammerhead sharks were analysed to characterise their lipid and fatty acid profiles. Samples were compared both between and within species, but there were no significant differences in total lipids for either comparison, although much greater total amounts were found in the liver samples. Within the individual fatty acids, the only significant differences were greater amounts of 22:6n-3, total n-3 polyunsaturates and total polyunsaturates in smooth, when compared to scalloped, hammerhead liver. This may reflect the more wide spread distribution of this species into cooler waters. Within both species the liver levels of the same fatty acid fractions decreased from spring to summer, which may correlate with changes in fatty acid profile to adapt to any differences in amount or species of prey consumed, or other considerations, eg. buoyancy, however there was no data to clarify this.

## 1. Introduction

The Indian Ocean coastline of South Africa is home to many species of shark, and some of these have the reputation of being dangerous to humans (Cliff and Dudley 1992; Cliff and Wilson 1986). As a direct result of shark attack the Natal Sharks Board (NSB), later KwaZulu-Natal Sharks Board (KZNSB), was established to protect people utilizing the surf zone. The main mechanism employed to reduce the incidence of shark attack was the placing of nets off the coastline to catch large sharks (Cliff and Dudley 1992; Cliff and Wilson 1986). This practice has led to the development of a large database of the sharks most commonly found off the KwaZulu-Natal (KZN) coast (egs. Allen and Cliff 2000; Cliff 1995; De Bruyn et al. 2005; Dudley and Cliff 1993), and at the same time, allowed for the collection of research materials from sharks caught in the nets.

Unlike mammals, that predominantly synthesise and utilise triacylglycerols (TAG) for energy storage and production, elasmobranchs exhibit a wider range of lipid classes that play roles in hepatic lipid storage and metabolism. Shallow water sharks exhibit a preponderance of TAG, alkyldiacylglycerols (ADAG) and squalene (Davidson, unpublished data), while deeper water sharks also incorporate wax esters (WE) (Ballantyne 1997). While hepatic storage of lipid is generally in the form of one of these classes, extra-hepatic tissues do not utilise them, or their fatty acids, directly to feed energy production, but rather rely on ketone (acetoacetate and β-hydroxybutyrate) production in the liver, and their subsequent export via the circulation (Ballantyne 1997, Sargent 1976). Thus, while in most other vertebrates ketones are produced as a response to reduced availability of dietary input (Newsholme and Leech 1983), in elasmobranchs they are produced under normal circumstances as a primary energy source for extra-hepatic tissues (Chamberlin and Ballantyne 1992; Moon and Mommsen 1987; Moyes et al. 1990; Singer and Ballantyne 1989). In contrast they do not provide the primary energy source for the liver as the capacity for hepatic ketone oxidation has been shown to be limiting (Ballantyne and Moon 1985, 1986; Mommsen and Moon 1987; Moyes et al. 1986).

However, hepatic lipids also serve a function in buoyancy control in elasmobranchs, and it has been suggested that the proportions of WE, ADAG, TAG and squalene are moderated to achieve optimal buoyancy (Craik 1978; Sargent 1976; Van Vleet et al. 1984). This is feasible as the different lipid classes exhibit different average specific gravities (Malins and Barone 1970).

Shark liver oils have been reported to be rich in polyunsaturated fatty acids, especially the n3 moieties (Davidson and Cliff 2003, 2011; Davidson et al. 2011a, [b]; Nichols et al. 1998; Nichols et al. 2001; Wetherbee and Nichols 2000). Data on the liver fatty acids of sharks that occur around Africa is limited (Banjo 1979; Davidson and Cliff 2003, 2011; Davidson et al. 2011a, [b]; Jayasinghe et al. 2003; Peyronel et al. 1984), although the liver oil from certain West African sharks has been shown to be composed predominantly of TAG, ADAG and hydrocarbons (Sargent et al. 1973).

Given the dearth of data on elasmobranchs from this region and as part of a large project to quantify the lipids of South African shark species, in this study we report the results of analyses of the fatty acids found in the total lipid fractions from the livers of two of the mid-sized shark species resident in South African coastal waters - the scalloped hammerhead (*Sphyrna zygaena*) and the smooth hammerhead (*Sphyrna lewini*).

## 2. Materials and methods

Details of the netting operation to protect the popular beaches of KZN used extensively for water-based activities are given in Cliff and Dudley (1992). All sharks found dead in the nets were taken to the laboratories of the KZNSB, where they were identified by expert staff, and the carcasses either dissected immediately, or frozen for future dissection. During dissection approximately 20 g samples were collected from the liver (upper portion of the left lobe), heart (base of the ventricle) and abdominal muscle (adjacent to the left pectoral fin), placed into labeled glass vials and frozen at −20°C. Previously, samples had been taken from three distinct and separate sites in livers of other sharks and no differences in total lipid or fatty acid profile demonstrated irrespective of sample site (Davidson et al. 2011b). Samples were collected, either by the first author or by KZNSB staff, from individual sharks between 2002 and 2007. Since all samples were taken post mortem and no sharks were purposely killed for this study there were no ethical considerations and ethical clearance was not deemed necessary by the Animal Ethics Committee of the University of the Witwatersrand, Johannesburg, South Africa.

All samples were transported frozen, then defrosted and analysed at the University of the Witwatersrand Medical School within six months of capture of the shark. The samples were weighed and the lipids then extracted according to Bligh and Dyer (1959). The extracts were reduced in volume and made to 20 ml with chloroform for storage. A one ml aliquot of each extract was then used to determine the lipid dry weight, and a further aliquot approximating to 20 mg of lipid was then transmethylated using 10% acetyl chloride in methanol to prepare the fatty acid methyl esters (FAME) (Christie 2003). These were then extracted into hexane, and separated using a Varian 3400 gas chromatograph with 4270 integrator and a 10% SP2330 on Chromosorb WAW 6′ × 1/8″ packed column using helium as carrier gas and run isothermally at 195°C with flame ionization detection (FID). FAME were identified by comparison with authentic standards purchased from Sigma-Aldrich, USA, (product numbers 18912, 18913, 18917 and ME14) and the data compared using the t-test (Statistica 9 software package).

## 3. Results

Indicators of statistically significant differences are shown in the Tables by lower case and upper case letters immediately after the data. Lower case letters indicate differences between species, while upper case letters indicate differences within species. All statistically significant differences (p < 0.05) are also identified in the text.

The results are shown as ‘Pooled’, which is all data irrespective of season or region; ‘Spring’, which is the southern hemisphere spring (September, October, November); ‘Summer’, which is summer (December, January, February); ‘Autumn’, which is autumn (March, April, May); ‘Winter’, which is winter (June, July, August); ‘North’, which is the northern region of the netted area; ‘Mid’, which is the mid region; and ‘South’, which is the southern region. The three regions represented the entire coastline of KZN.

The demographical distribution of the individual sharks of each species can be seen in Table 1. Comparison of the lipid and fatty acid data within or between year or month did not indicate any significant differences, although the sample numbers were low when the data was sub-divided on this basis. It is possible differences might appear with a larger sample size.

**Table 1:** **Demographic data of the smooth and scalloped hammerhead sharks**

Scalloped							
Year caught	Month	Age	Sex	Season	Region	Mass (kg)	Length (cm)
2002	12	A	F	SU	N	102	194
2003	01	J	F	SU	N	29	119
	09	A	M	SP	S	140	220
2004	09	A	F	SP	S	96	200
	10	A	F	SP	M	83	186
	10	A	M	SP	M	98	196
	11	A	M	SP	M	96	197
	12	A	F	SU	M	120	211
	12	A	M	SU	M	88	178
2005	01	A	M	SU	N	92	196
	01	A	F	SU	N	136	220
	10	J	F	SP	S	30	121
2006	02	A	F	SU	N	148	215
	09	A	F	SP	S	165	245
**Smooth**							
**Year caught**	**Month**	**Age**	**Sex**	**Season**	**Region**	**Mass (kg)**	**Length (cm)**
2002	09	A	F	SP	S	88	165
2003	03	A	M	AU	M	96	176
	08	J	M	WI	S	21	98
	08	A	F	WI	M	76	118
	09	A	M	SP	S	120	208
2004	01	A	M	SU	M	110	197
	01	A	F	SU	M	153	234
	03	A	F	AU	M	127	216
	04	A	M	AU	M	111	199
	08	J	F	WI	S	24	99
	09	A	F	SP	M	94	192
	11	J	M	SP	M	36	121
	12	A	F	SU	M	104	194
2005	01	A	F	SU	M	89	187
	02	A	M	SU	M	115	211
	06	A	M	WI	M	87	178
	07	A	F	WI	M	97	178
	08	A	M	WI	M	114	206
	09	A	F	SP	S	123	212

The results of the analysis of the total lipid content of the three tissue types can be seen in Table 2. There were no statistically significant differences between species, season or geographical region. However, there were trends within the liver samples. Comparing scalloped and smooth hammerhead, as pooled data irrespective of season or region, there were greater amounts of total lipid in the smooth hammerhead livers (scalloped 96 mg g^−1^ and smooth 131 mg g^−1^). This pattern was not repeated when Spring samples were compared with Summer. Scalloped hammerhead samples showed a decrease in total lipid from Spring to Summer (123 mg g^−1^ and 80 mg g^−1^), while smooth hammerhead showed the reverse (96 mg g^−1^ and 120 mg g^−1^) and the increase continued through Autumn and Winter (159 mg g^−1^ and 162 mg g^−1^). Comparing between region for each species, smooth hammerhead samples showed greater amounts of total lipid in samples from the Mid and South (143 mg g^−1^ and 70 mg g^−1^) compared to scalloped hammerhead samples (91 mg g^−1^ and 59 mg g^−1^). There were no smooth hammerhead samples from the North thus no comparison was possible. Scalloped hammerhead liver samples showed greater total lipid in samples from the North compared to both Mid and South (136 mg g^−1^, 91 mg g^−1^ and 59 mg g^−1^).

**Table 2:** **Comparison of the total lipid of the scalloped and smooth hammerhead samples**

Lipid	Scalloped (total n = 14)			Smooth (total n = 19)		
mg g ^−1^	Liver (n = 14)	Heart (n = 12)	Muscle (n = 9)	Liver (n = 19)	Heart (n = 10)	Muscle (n = 10)
Pooled	96 ± 21	3 ± 1	2 ± 1	131 ± 38	4 ± 3	4 ± 3
Spring	123 ± 33	3 ± 1	3 ± 2	96 ± 29	1 ± 2	2 ± 2
Summer	80 ± 26	4 ± 1	2 ± 1	120 ± 30	6 ± 4	5 ± 3
Autumn	-	-	-	159 ± 13	7	7
Winter	-	-	-	162 ± 19	6 ± 5	6 ± 1
North	136 ± 40	3 ± 1	2 ± 2	-	-	-
Mid	91 ± 35	3 ± 1	1 ± 1	143 ± 30	5 ± 4	4 ± 3
South	59 ± 21	3 ± 1	3 ± 2	70 ± 24	2 ± 1	2 ± 2

Tables 3, 4 and 5 show the fatty acid profiles of the liver, heart and abdominal muscle samples, respectively, expressed as percentages of the total fatty acid methyl esters in all cases. Each Table shows the pooled fatty acid percentages irrespective of season or region, as well as taking such into consideration. Tables 4 and 5 include the results for smooth hammerhead in Autumn, but no statistical analysis was possible as only one sample was obtained. This data is included for comparative purposes.

**Table 3:** **Comparison of the fatty acids of the scalloped and smooth hammerhead liver samples**

Fatty acid	Scalloped						Smooth						
µ ± SD	Pooled n = 14	Spring n = 7	Summer n = 7	North n = 5	Mid n = 5	South n = 4	Pooled n = 19	Spring n = 5	Summer n = 5	Autumn n = 3	Winter n = 6	Mid n = 14	South n = 5
14:0	2.62 ± 1.29	3.12 ± 1.72	2.19 ± 0.69	3.30 ± 0.66	2.90 ± 0.71	1.78 ± 0.68	2.40 ± 0.94	0.59 ± 0.15	2.64 ± 0.64	1.75 ± 0.23	2.53 ± 0.23	2.60 ± 0.25	1.37 ± 0.24
16:0	17.91 ± 2.02	14.26 ± 2.41	20.46 ± 2.93	17.72 ± 3.38	19.56 ± 1.00	17.11 ± 2.07	16.58 ± 1.73	19.02 ± 1.10	15.43 ± 1.12	14.29 ± 0.80	17.19 ± 0.41	17.37 ± 2.64	12.35 ± 2.80
18:0	6.74 ± 1.15	4.49 ± 1.88	8.11 ± 1.55	5.99 ± 0.53	7.35 ± 1.74	7.12 ± 1.95	6.10 ± 0.71	5.37 ± 1.19	5.67 ± 1.90	7.69 ± 1.99	6.16 ± 0.25	5.95 ± 1.16	6.88 ± 1.43
TotalSFA	27.39 ± 3.17	21.96 ± 2.27	30.92 ± 4.42	27.19 ± 2.08	29.89 ± 2.62	26.08 ± 2.97	25.12 ± 1.71	25.10 ± 1.99	23.56 ± 2.61	23.74 ± 2.58	25.89 ± 0.70	25.93 ± 1.23	20.79 ± 3.49
14:1n7	0.41 ± 0.34	0.21 ± 0.24	0.52 ± 0.29	0.29 ± 0.21	0.51 ± 0.17	0.48 ± 0.22	0.51 ± 0.19	0.12 ± 0.11	0.64 ± 0.28	0.35 ± 0.02	0.36 ± 0.06	0.56 ± 0.123	0.21 ± 0.10
16:1n9	10.72 ± 2.39	14.07 ± 2.80	7.76 ± 1.71	12.47 ± 1.13	11.83 ± 2.28	8.31 ± 1.91	5.77 ± 1.78	3.84 ± 1.89	5.43 ± 1.16	6.28 ± 0.60	6.96 ± 0.37	6.06 ± 1.91	4.25 ± 1.06
18:1n9	20.48 ± 2.90	20.13 ± 1.41	21.16 ± 2.93	17.81 ± 2.69	19.63 ± 2.28	23.66 ± 2.68	13.60 ± 1.19	16.40 ± 1.55	12.55 ± 0.76	12.41 ± 0.47	13.23 ± 0.21	13.69 ± 2.26	13.16 ± 2.18
TotalMUFA	31.60 ± 2.96	34.38 ± 2.05	29.44 ± 2.65	30.57 ± 1.40	31.97 ± 1.84	32.41 ± 2.18	19.88 ± 2.07	20.36 ± 1.88	18.50 ± 2.16	19.04 ± 0.80	20.55 ± 0.61	20.30 ± 3.03	17.62 ± 2.58
16:2n6	0.54 ± 0.67	0.22 ± 0.34	0.72 ± 0.81	0.40 ± 0.19	0.73 ± 0.42	0.57 ± 0.28	0.53 ± 0.27	0.21 ± 0.10	0.60 ± 0.24	0.53 ± 0.03	0.57 ± 0.04	0.56 ± 0.17	0.36 ± 0.13
18:2n6	0.48 ± 0.58	0.32 ± 0.49	0.72 ± 0.65	0.57 ± 0.18	0.21 ± 0.17	0.54 ± 0.15	1.27 ± 0.24	0.29 ± 0.10	1.46 ± 1.00	1.02 ± 0.14	1.04 ± 0.05	1.35 ± 0.34	0.86 ± 0.28
20:2n6	0.16 ± 0.21	0.14 ± 0.22	0.20 ± 0.22	0.25 ± 0.12	0.11 ± 0.05	0.09 ± 0.01	0.46 ± 0.31	0.19 ± 0.16	0.40 ± 0.31	0.16 ± 0.27	0.48 ± 0.53	0.24 ± 0.09	1.61 ± 0.99
20:3n6	0.04 ± 0.16	0.00	0.00	0.00	0.19 ± 0.12	0.00	0.08 ± 0.07	0.00	0.08 ± 0.01	0.15 ± 0.08	0.19 ± 0.09	0.10 ± 0.07	0.00
20:4n6	3.31 ± 0.58	2.78 ± 0.99	3.76 ± 0.19	3.02 ± 0.87	3.17 ± 0.34	3.69 ± 1.15	2.88 ± 0.35	3.44 ± 0.95	2.60 ± 0.58	3.40 ± 0.57	2.97 ± 0.06	2.59 ± 0.26	4.46 ± 1.89
22:4n6	0.89 ± 0.12	0.36 ± 0.50	1.38 ± 0.46	0.50 ± 0.40	0.62 ± 0.13	1.43 ± 1.17	0.59 ± 0.30	0.30 ± 0.28	0.55 ± 0.12	0.78 ± 0.18	0.81 ± 0.10	0.58 ± 0.12	0.66 ± 0.09
22:5n6	1.61 ± 0.09	0.95 ± 0.19	2.18 ± 0.68	1.30 ± 0.77	1.42 ± 0.19	2.03 ± 1.52	1.59 ± 0.24	1.80 ± 0.11	1.50 ± 0.54	1.59 ± 0.09	1.54 ± 0.08	1.22 ± 0.30	3.54 ± 0.97
Totaln6PUFA	7.06 ± 1.94	4.83 ± 0.47	9.97 ± 1.49	6.15 ± 0.95	6.44 ± 0.33	8.34 ± 1.29	7.38 ± 1.79	6.14 ± 1.26	6.97 ± 0.95	7.62 ± 0.56	7.59 ± 0.62	6.64 ± 1.33	11.35 ± 1.07
18:3n3	4.06 ± 0.71	3.60 ± 0.91	4.74 ± 0.77	2.82 ± 0.45	3.03 ± 0.70	5.93 ± 1.00	1.49 ± 0.11	1.57 ± 0.01	1.53 ± 0.05	1.38 ± 0.47	1.23 ± 0.19	1.30 ± 0.47	2.50 ± 0.18
18:4n3	0.08 ± 0.15	0.06 ± 0.13	0.13 ± 0.17	0.13 ± 0.14	0.04 ± 0.01	0.07 ± 0.01	0.18 ± 0.15	0.06 ± 0.01	0.16 ± 0.03	0.00	0.87 ± 0.19	0.21 ± 0.19	0.09 ± 0.11
20:3n3	0.06 ± 0.08	0.05 ± 0.07	0.08 ± 0.09	0.09 ± 0.07	0.04 ± 0.01	0.03 ± 0.01	0.10 ± 0.04	0.09 ± 0.03	0.10 ± 0.10	0.12 ± 0.01	0.13 ± 0.03	0.09 ± 0.04	0.15 ± 0.13
20:5n3	6.13 ± 1.82	9.19 ± 0.80	3.41 ± 1.06	8.00 ± 1.46	6.70 ± 031	3.91 ± 1.04	6.81 ± 0.59	4.17 ± 01.97	6.21 ± 1.06	7.16 ± 0.65	9.62 ± 0.29	7.47 ± 1.17	3.32 ± 0.84
22:5n3	1.62 ± 0.15	1.07 ± 044	2.06 ± 0.67	1.51 ± 0.09	1.73 ± 0.21	1.66 ± 0.49	2.46 ± 0.29	1.55 ± 0.70	2.27 ± 0.14	3.33 ± 0.07	3.34 ± 0.16	2.51 ± 1.22	2.23 ± 0.93
22:6n3	20.70 ± 3.71a	24.38 ± 1.21bA	18.26 ± 2.28A	22.83 ± 1.01	19.02 ± 1.43c	19.58 ± 2.39d	35.28 ± 3.05a	40.30 ± 2.11bA	32.77 ± 2.66A	37.06 ± 2.26	30.25 ± 1.28	34.48 ± 3.70c	39.58 ± 3.64d
Totaln3PUFA	32.65 ± 2.19a	38.35 ± 2.01bA	28.67 ± 2.95A	35.37 ± 2.51	30.56 ± 3.91c	31.17 ± 3.54d	46.28 ± 2.80a	47.63 ± 3.60bA	42.67 ± 2.19A	49.04 ± 2.28	45.01 ± 1.16	45.99 ± 2.35c	47.81 ± 3.99d
TotalPUFA	39.67 ± 2.93a	43.10 ± 1.91bA	37.64 ± 1.21A	41.43 ± 2.93	37.01 ± 2.24c	39.52 ± 2.05d	53.66 ± 1.07a	53.77 ± 2.66bA	49.55 ± 3.09A	56.67 ± 2.63	52.60 ± 0.98	52.63 ± 2.91c	59.16 ± 5.64d

Data shown as mean ± SD.

‘n’ refers to the number of individual fish sampled in all cases.

‘Pooled’ = all sample data pooled irrespective of season or region.

‘TotalSFA’ = total saturated fatty acids.

‘TotalMUFA’ = total monounsaturated fatty acids.

‘TotalPUFA’ = total polyunsaturated fatty acids.

‘Totaln-6PUFA’ = total n-6 polyunsaturated fatty acids.

‘Totaln-3PUFA’ = total n-3 polyunsaturated fatty acids.

Seasons: ‘SP’ = spring; ‘SU’ = summer; ‘AU’ = autumn; ‘WI’ = winter.

Regions: ‘N’ = north; ‘M’ = mid; ‘S’ = south.

‘a’ = significant differences between smooth and scalloped data sets labelled ‘a’.

‘b’ = significant differences between smooth and scalloped data sets labelled ‘b’.

‘c’ = significant differences between smooth and scalloped data sets labelled ‘c’.

‘d’ = significant differences between smooth and scalloped data sets labelled ‘d’.

‘A’ = significant differences between Spring and Summer data sets labelled ‘A’.

**Table 4:** **Comparison of the fatty acids of the scalloped and smooth hammerhead heart samples**

Fatty acid	Scalloped						Smooth						
µ ± SD	Pooled n = 12	Spring n = 6	Summer n = 6	North n = 6	Mid n = 3	South n = 3	Pooled n = 10	Spring n = 3	Summer n = 3	Autumn n = 1	Winter n = 3	Mid n = 7	South n = 3
14:0	0.92 ± 0.10	1.02 ± 0.35	0.87 ± 0.37	0.85 ± 0.55	0.65 ± 0.16	1.35 ± 0.89	0.91 ± 0.42	0.64 ± 0.04	0.77 ± 0.43	1.00	1.44 ± 0.49	1.00 ± 0.51	0.78 ± 0.26
16:0	16.43 ± 2.80	15.57 ± 1.85	17.37 ± 2.19	15.63 ± 1.70	15.02 ± 1.17	19.43 ± 7.25	13.99 ± 1.50	13.61 ± 0.83	13.20 ± 2.04	13.43	15.64 ± 1.65	13.82 ± 1.95	14.27 ± 0.35
18:0	16.90 ± 2.97	14.45 ± 1.35	18.89 ± 2.39	15.45 ± 6.15	17.77 ± 2.38	18.93 ± 4.73	18.01 ± 4.56	21.21 ± 2.24	19.89 ± 0.91	15.56	12.54 ± 2.26	15.50 ± 3.49	22.18 ± 2.58
TotalSFA	34.23 ± 2.35	31.05 ± 1.75	37.07 ± 2.90	31.88 ± 6.28	33.45 ± 3.62	39.71 ± 11.09	32.91 ± 3.87	35.46 ± 3.04	33.85 ± 1.57	29.99	29.62 ± 0.12	30.32 ± 1.36	37.23 ± 2.00
14:1n7	1.41 ± 0.53	1.23 ± 0.42	1.70 ± 0.50	1.42 ± 1.18	1.61 ± 0.91	1.21 ± 0.59	0.59 ± 0.30	0.77 ± 0.16	0.45 ± 0.64	0.45	0.52 ± 0.05	0.66 ± 0.23	0.46 ± 0.40
16:1n9	2.64 ± 0.99	3.94 ± 0.46	1.55 ± 0.54	3.04 ± 2.17	1.95 ± 0.66	2.52 ± 2.88	3.01 ± 2.23	1.34 ± 0.37	1.86 ± 1.22	5.42	5.46 ± 2.04	3.84 ± 2.45	1.62 ± 0.96
18:1n9	17.21 ± 2.00	21.10 ± 1.94	14.52 ± 0.86	20.40 ± 0.66	14.53 ± 0.66	13.49 ± 2.33	12.75 ± 2.25	11.69 ± 1.40	11.68 ± 0.65	13.97	14.80 ± 3.95	13.00 ± 2.92	12.34 ± 0.57
TotalMUFA	21.59 ± 2.85	26.28 ± 1.82	18.44 ± 1.65	25.53 ± 11.59	18.10 ± 1.05	17.21 ± 0.21	16.34 ± 4.15	13.79 ± 0.92	13.99 ± 1.24	19.84	20.77 ± 5.94	17.50 ± 5.04	14.42 ± 0.71
16:2n6	0.68 ± 0.34	0.52 ± 0.53	0.74 ± 0.60	0.83 ± 0.49	1.07 ± 0.18	0.00	0.73 ± 0.55	1.18 ± 0.13	0.65 ± 0.91	0.00	0.53 ± 0.02	0.67 ± 0.50	0.83 ± 0.72
18:2n6	0.67 ± 0.37	0.82 ± 0.60	0.67 ± 0.36	0.68 ± 0.63	0.59 ± 0.51	0.75 ± 0.71	0.56 ± 0.27	0.49 ± 0.017	0.67 ± 0.08	0.89	0.39 ± 0.49	0.52 ± 0.34	0.62 ± 0.13
20:2n6	0.00	0.00	0.00	0.00	0.00	0.00	0.11 ± 0.25	0.05 ± 0.03	0.00	0.70	0.00	0.17 ± 0.30	0.00
20:3n6	0.00	0.00	0.00	0.00	0.00	0.00	0.06 ± 0.07	0.07 ± 0.02	0.00	0.12	0.06 ± 0.04	0.09 ± 0.08	0.00
20:4n6	8.31 ± 0.92	6.89 ± 1.07	9.28 ± 1.08	7.77 ± 2.10	9.66 ± 0.02	8.06 ± 2.36	7.84 ± 2.04	8.96 ± 0.41	9.38 ± 1.34	5.76	5.68 ± 1.07	6.96 ± 2.09	9.31 ± 0.82
22:4n6	1.02 ± 0.72	1.15 ± 0.78	1.08 ± 0.26	1.28 ± 0.67	1.05 ± 0.95	0.46 ± 0.10	1.34 ± 1.32	2.43 ± 0.50	0.96 ± 1.35	0.59	0.48 ± 0.15	0.84 ± 0.61	2.17 ± 1.93
22:5n6	1.14 ± 0.85	0.72 ± 0.27	1.46 ± 0.29	1.11 ± 0.19	0.91 ± 0.32	1.43 ± 0.36	0.79 ± 0.61	0.48 ± 0.14	1.05 ± 0.83	0.84	0.96 ± 0.01	0.93 ± 0.35	0.55 ± 0.95
Totaln6PUFA	11.82 ± 1.99	10.10 ± 1.50	13.21 ± 2.14	11.67 ± 1.57	13.27 ± 1.04	10.67 ± 1.34	11.43 ± 2.08	13.65 ± 2.15	12.70 ± 2.69	8.90	8.09 ± 0.76	10.19 ± 1.93	13.49 ± 2.41
18:3n3	2.09 ± 0.86	3.52 ± 1.23	1.10 ± 0.30	3.16 ± 1.89	0.92 ± 0.27	1.11 ± 0.32	0.72 ± 0.34	0.77 ± 0.12	0.27 ± 0.18	1.07	0.92 ± 0.12	0.87 ± 0.20	0.47 ± 0.01
18:4n3	0.12 ± 0.10	0.11 ± 0.10	0.15 ± 0.07	0.13 ± 0.11	0.21 ± 0.10	0.00	0.14 ± 0.10	0.18 ± 0.08	0.13 ± 0.08	0.00	0.17 ± 0.01	0.14 ± 0.01	0.15 ± 0.03
20:3n3	0.15 ± 0.10	0.17 ± 0.13	0.16 ± 0.09	0.20 ± 0.12	0.20 ± 0.11	0.00	0.28 ± 0.13	0.54 ± 0.38	0.23 ± 0.02	0.05	0.06 ± 0.04	0.16 ± 0.07	0.48 ± 0.16
20:5n3	4.31 ± 0.80 g	4.66 ± 0.47	3.88 ± 0.88	3.08 ± 1.04	4.47 ± 0.19hB	6.60 ± 1.09B	7.65 ± 1.33 g	6.88 ± 0.67	7.26 ± 0.12	7.92	9.07 ± 0.75	8.43 ± 0.86hB	6.35 ± 0.78B
22:5n3	2.10 ± 0.12	2.10 ± 0.46	1.95 ± 0.33	1.85 ± 0.39	2.45 ± 0.35	2.26 ± 0.90	2.95 ± 0.75	2.99 ± 0.40	2.42 ± 0.30	3.05	3.36 ± 0.65	3.31 ± 0.73	2.35 ± 0.23
22:6n3	21.22 ± 3.87 g	20.56 ± 2.24	21.44 ± 2.17	21.04 ± 2.24	23.20 ± 0.07hB	19.60 ± 2.83B	25.71 ± 2.11 g	23.29 ± 3.74	25.23 ± 2.25	29.83	27.76 ± 1.94	27.78 ± 2.73hB	22.26 ± 3.95B
Totaln3PUFA	29.98 ± 3.60 g	31.11 ± 3.06	28.68 ± 3.98	29.45 ± 2.32	31.45 ± 3.31hB	29.56 ± 1.51B	37.30 ± 2.50 g	34.27 ± 2.42	35.53 ± 0.94	41.92	41.33 ± 3.56	40.45 ± 3.65hB	32.06 ± 3.61B
TotalPUFA	41.80 ± 3.20 g	41.21 ± 2.04	41.88 ± 2.85	41.12 ± 2.63	44.72 ± 3.16hB	40.23 ± 1.56B	48.73 ± 3.71 g	47.92 ± 1.36	48.23 ± 1.75	50.82	49.42 ± 2.32	50.64 ± 3.34hB	45.55 ± 1.26B

Data shown as mean ± SD.

‘n’ refers to the number of individual fish sampled in all cases.

‘Pooled’ = all sample data pooled irrespective of season or region.

‘TotalSFA’ = total saturated fatty acids.

‘TotalMUFA’ = total monounsaturated fatty acids.

‘TotalPUFA’ = total polyunsaturated fatty acids.

‘Totaln-6PUFA’ = total n-6 polyunsaturated fatty acids.

‘Totaln-3PUFA’ = total n-3 polyunsaturated fatty acids.

Seasons: ‘SP’ = spring; ‘SU’ = summer; ‘AU’ = autumn; ‘WI’ = winter.

Regions: ‘N’ = north; ‘M’ = mid; ‘S’ = south.

‘a’ = significant differences between smooth and scalloped data sets labelled ‘a’.

‘b’ = significant differences between smooth and scalloped data sets labelled ‘b’.

‘c’ = significant differences between smooth and scalloped data sets labelled ‘c’.

‘d’ = significant differences between smooth and scalloped data sets labelled ‘d’.

‘g’ = significant differences between smooth and scalloped data sets labelled ‘g’.

‘h’ = significant differences between smooth and scalloped data sets labelled ‘h’.

‘B’ = significant differences between Mid and South data sets labelled ‘B’.

**Table 5:** **Comparison of the fatty acids of the scalloped and smooth hammerhead abdominal muscle samples**

Fatty acid	Scalloped						Smooth						
µ ± SD	Pooled n = 9	Spring n = 3	Summer n = 6	North n = 3	Mid n = 3	South n = 3	Pooled n = 10	Spring n = 3	Summer n = 3	Autumn n = 1	Winter n = 3	Mid n = 7	South n = 3
14:0	0.86 ± 0.17	0.98 ± 0.34	0.72 ± 0.18	0.58 ± 0.13	0.73 ± 0.20	1.27 ± 0.17	1.47 ± 0.38	0.60 ± 0.04	1.83 ± 0.28	1.45	2.25 ± 0.40	1.47 ± 0.36	1.09 ± 0.53
16:0	13.52 ± 2.09	12.43 ± 1.62	13.50 ± 1.09	11.60 ± 0.38	13.79 ± 2.63	15.18 ± 0.77	15.77 ± 1.77	13.35 ± 0.49	17.08 ± 2.46	14.44	18.08 ± 1.41	15.77 ± 1.67	15.28 ± 1.52
18:0	16.75 ± 1.05	16.54 ± 1.20	17.72 ± 1.66	17.07 ± 1.83	17.92 ± 1.97	15.27 ± 2.95	14.07 ± 1.65	18.82 ± 2.55	13.29 ± 1.88	10.07	10.10 ± 0.25	14.07 ± 1.44	18.02 ± 2.64
TotalSFA	31.14 ± 2.73	29.97 ± 1.37	31.94 ± 3.30	29.25 ± 1.57	32.43 ± 1.25	31.74 ± 2.16	31.42 ± 2.86	32.78 ± 3.16	32.21 ± 2.33	27.00	30.42 ± 2.35	31.42 ± 1.82	34.40 ± 2.12
14:1n7	5.90 ± 3.61	6.61 ± 0.76	6.21 ± 1.17	9.07 ± 1.80	6.40 ± 1.55	2.22 ± 0.47	3.90 ± 1.53	6.10 ± 0.86	3.30 ± 0.61	2.19	2.33 ± 0.04	3.90 ± 1.42	4.84 ± 1.98
16:1n9	1.36 ± 0.52	1.89 ± 0.58	0.77 ± 0.28	0.84 ± 0.12	1.06 ± 0.20	2.18 ± 0.74	3.64 ± 1.87	1.14 ± 0.23	5.10 ± 1.30	4.99	4.50 ± 0.54	3.64 ± 1.10	2.49 ± 0.42
18:1n9	13.18 ± 1.98	13.83 ± 1.34	13.66 ± 1.91	13.28 ± 1.26	12.68 ± 1.85	13.59 ± 1.33	15.75 ± 1.50	11.97 ± 1.50	20.00 ± 2.65	12.66	16.58 ± 2.12	15.75 ± 1.22	13.55 ± 1.25
TotalMUFA	20.44 ± 2.14	22.33 ± 1.30	20.64 ± 2.36	23.20 ± 1.36	20.14 ± 2.27	17.99 ± 1.28	23.28 ± 1.41	19.22 ± 2.19	28.40 ± 1.20	19.84	23.41 ± 1.63	23.28 ± 1.38	20.87 ± 0.17
16:2n6	0.53 ± 0.22	0.54 ± 0.19	0.42 ± 0.22	0.86 ± 0.06	0.72 ± 0.24	0.00	0.59 ± 0.26	0.61 ± 0.06	0.32 ± 0.13	0.89	0.84 ± 0.19	0.59 ± 0.25	0.40 ± 0.15
18:2n6	0.81 ± 0.25	0.85 ± 0.15	0.96 ± 0.18	0.88 ± 0.10	0.54 ± 0.38	1.01 ± 0.17	1.43 ± 0.26	0.66 ± 0.18	3.05 ± 1.77	0.00	0.86 ± 0.02	1.43 ± 0.81	0.89 ± 0.25
20:2n6	0.00	0.00	0.00	0.00	0.00	0.00	0.00	0.00	0.00	0.00	0.00	0.00	0.00
20:3n6	0.00	0.00	0.00	0.00	0.00	0.00	0.21 ± 0.05	0.07 ± 0.02	0.11 ± 0.08	0.00	0.69 ± 0.22	0.21 ± 0.19	0.00
20:4n6	7.63 ± 0.61	6.90 ± 0.61	8.04 ± 0.87	8.39 ± 1.30	7.99 ± 0.47	6.52 ± 0.90	5.63 ± 1.05	7.58 ± 0.73	5.01 ± 1.51	4.89	4.00 ± 0.20	5.63 ± 0.84	6.47 ± 0.59
22:4n6	3.95 ± 0.23	3.37 ± 0.60	4.43 ± 0.33	4.26 ± 0.61	3.68 ± 0.02	3.92 ± 0.32	2.11 ± 0.37	3.36 ± 0.32	1.99 ± 0.21	1.11	0.92 ± 0.22	2.11 ± 0.99	3.13 ± 0.66
22:5n6	1.53 ± 0.02	1.15 ± 0.18	1.41 ± 0.28	1.31 ± 0.26	1.65 ± 0.06	1.63 ± 0.68	1.34 ± 0.50	1.21 ± 0.13	1.47 ± 0.19	1.61	1.19 ± 0.39	1.34 ± 0.33	1.61 ± 0.44
Totaln6PUFA	14.45 ± 1.69	12.81 ± 1.01	15.26 ± 1.71	15.70 ± 1.89	14.58 ± 1.30	13.08 ± 1.29	11.31 ± 2.87	13.49 ± 1.10	11.95 ± 1.94	8.50	8.48 ± 0.77	11.31 ± 1.59	12.50 ± 1.62
18:3n3	0.86 ± 0.18	0.96 ± 0.18	0.88 ± 0.25	0.80 ± 0.26	0.71 ± 0.17	1.07 ± 0.13	1.37 ± 0.36	0.66 ± 0.12	1.62 ± 0.28	1.53	1.99 ± 0.30	1.37 ± 0.37	1.50 ± 0.33
18:4n3	0.09 ± 0.02	0.14 ± 0.04	0.09 ± 0.06	0.17 ± 0.03	0.10 ± 0.09	0.00	0.08 ± 0.03	0.16 ± 0.05	0.04 ± 0.01	0.00	0.00	0.08 ± 0.01	0.09 ± 0.05
20:3n3	0.18 ± 0.07	0.25 ± 0.09	0.20 ± 0.08	0.33 ± 0.04	0.21 ± 0.15	0.00	0.19 ± 0.01	0.37 ± 0.15	0.13 ± 0.09	0.00	0.10 ± 0.05	0.19 ± 0.03	0.29 ± 0.17
20:5n3	3.81 ± 0.66	4.18 ± 0.81	3.20 ± 0.59	3.34 ± 0.57	3.28 ± 0.88	4.80 ± 1.73	4.91 ± 0.96	4.54 ± 0.31	3.24 ± 0.35	7.37	6.75 ± 1.48	4.91 ± 0.15	3.66 ± 0.56
22:5n3	4.17 ± 0.67	4.81 ± 1.52	3.36 ± 0.77	3.41 ± 0.47	4.35 ± 0.76	4.74 ± 1.93	4.51 ± 0.78	5.86 ± 1.05	3.47 ± 0.84	4.49	4.04 ± 0.94	4.51 ± 0.16	4.45 ± 0.95
22:6n3	22.30 ± 1.91	23.06 ± 3.03	21.22 ± 2.00	23.03 ± 3.22	22.45 ± 1.46	21.41 ± 2.39	21.32 ± 1.44	22.02 ± 3.92	16.31 ± 1.03	31.29	22.80 ± 2.51	21.32 ± 2.23	20.13 ± 2.33
Totaln3PUFA	32.50 ± 1.53	35.84 ± 1.11	28.95 ± 3.48	31.02 ± 2.04	31.10 ± 2.21	35.36 ± 3.67	32.26 ± 1.30	33.29 ± 2.53	24.82 ± 1.37	44.68	35.66 ± 2.68	32.26 ± 2.68	30.11 ± 2.49
TotalPUFA	46.95 ± 1.70	48.65 ± 3.29	44.21 ± 3.01	46.72 ± 1.91	45.68 ± 3.46	48.44 ± 3.39	43.57 ± 1.41	46.78 ± 3.45	36.77 ± 2.95	53.18	44.14 ± 3.45	43.57 ± 2.59	42.61 ± 3.05

Data shown as mean ± SD.

‘n’ refers to the number of individual fish sampled in all cases.

‘Pooled’ = all sample data pooled irrespective of season or region.

‘TotalSFA’ = total saturated fatty acids.

‘TotalMUFA’ = total monounsaturated fatty acids.

‘TotalPUFA’ = total polyunsaturated fatty acids.

‘Totaln-6PUFA’ = total n-6 polyunsaturated fatty acids.

‘Totaln-3PUFA’ = total n-3 polyunsaturated fatty acids.

Seasons: ‘SP’ = spring; ‘SU’ = summer; ‘AU’ = autumn; ‘WI’ = winter.

Regions: ‘N’ = north; ‘M’ = mid; ‘S’ = south.

Comparing scalloped liver with smooth, irrespective of season or region (Table 3) the smooth hammerhead livers showed greater levels of 22:6n-3 (46.28 vs. 20.70%, p < 0.05), Total n-3PUFA (46.28 vs. 32.65%, p < 0.05) and total PUFA (53.66 vs. 39.67%, p < 0.05). When comparing the Spring samples, the only significant differences were for 22:6n-3, total n-3PUFA and total PUFA (scalloped vs. smooth: 24.38, 38.35 and 43.10% vs. 40.30, 47.63 and 53.77%, p < 0.05). In parallel, when comparing the Mid and Southern regions, a similar pattern emerged with the only significant differences being for 22:6n-3, total n-3PUFA and total PUFA (scalloped Mid vs. smooth Mid: 19.02, 30.56 and 37.01% vs. 34.48, 45.99 and 52.63%, p < 0.05) (scalloped Southern vs. smooth Southern: 19.58, 31.17 and 39.52% vs. 39.58, 47.81 and 59.16%, p < 0.05). There were no significant differences for any of the SFA, MUFA or n-6PUFA.

Comparing between seasons, Table 3 shows the fatty acid profiles of samples obtained in Spring and Summer for the scalloped hammerhead and Spring, Summer, Autumn and Winter for the smooth hammerhead. The only significant differences were shown when comparing Spring with Summer in both species and, once more, were for 22:6n-3, total n-3PUFA and total PUFA (scalloped: 24.38 vs. 18.26%, p < 0.05, 38.35 vs. 28.67%, p < 0.05, and 43.10 vs. 37.64%, p < 0.05; smooth: 40.30 vs. 32.77%, p < 0.05, 47.63 vs. 42.67%, p < 0.05, and 53.77 vs. 49.55%, p < 0.05).

Table 4 shows the fatty acid profiles of the scalloped and smooth hammerhead heart samples. The only significant differences were obtained with 20:5n-3, 22:6n-3, total n-3PUFA and total PUFA, and only for the total, irrespective of season or region, and Mid. Smooth hammerhead samples showed increased levels of 20:5n-3, 22:6n-3, total n-3PUFA and total PUFA compared to scalloped hammerhead samples for both the total (7.65 vs. 4.32%, p < 0.05, 25.71 vs. 21.22%, p < 0.05, 37.30 vs. 29.98%, p < 0.05, and 48.78 vs. 41.80%, p < 0.05) and Mid (4.47 vs. 8.43%, p < 0.05, 27.78 vs. 23.20%, p < 0.05, 40.45 vs. 31.45%, p < 0.05, and 50.64 vs. 44.72%, p < 0.05).

Table 5 shows the results for the scalloped and smooth hammerhead abdominal muscle samples. There were no significant differences between any of the fatty acid fractions.

Of the 14 scalloped hammerheads, 12 were adult (five males and seven females) and two juveniles (both female). Of the 19 smooth hammerheads, 16 were adults (seven males and nine females) and three were juveniles (two males and one female). However, there were no differences demonstrated between males and females, or adults and juveniles, for either species (data not shown).

## 4. Discussion

Adipose tissue is absent from elasmobranchs, hence all lipid accumulation is achieved by moderating the hepatic storage of same (Ballantyne 1997). This would explain the considerable amounts of hepatic lipid, compared to both heart and abdominal muscle.

The low overall total lipid levels, as well as the minimal differences in heart and lack of any differences in abdominal muscle, argue that fatty acid metabolism in both tissues is not a major energy source and that the lipid present is largely in the form of metabolically inert structural lipids, predominantly cell membrane phosphoglycerides.

The relatively high levels of liver total lipid and the highly unsaturated nature of the fatty acids are indicative of the central role of the liver in lipid metabolism in elasmobranchs. The marine foodweb provides high levels of n-3PUFA, but relatively low levels of n-6PUFA. This may have contributed to the fatty acid profiles demonstrated, as the limited amounts of n-6PUFA would tend to be conserved, while the relatively abundant n-3PUFA would potentially be more variable as they are more readily available from the diet. This may at least partially explain the lack of any significant differences in n-6PUFA between the species, with all the differences being for n-3PUFA moieties. Both PUFA and MUFA have been shown to be important for buoyancy in elasmobranchs (Ballantyne 1997) and this may explain the relative constancy of MUFA composition within the liver fatty acid profiles.

Energy metabolism in elasmobranchs depends on fatty acid oxidation via β-oxidation in liver mitochondria to produce acetyl-CoA as the precursor for acetoacetate (ketone) synthesis. These ketones are then exported for use by extra-hepatic tissues (Baldridge 1970; Ballantyne 1997). This may be the reason for the SFA profiles also being relatively constant as they are the preferred fuel for β-oxidation (Ballantyne 1997).

Smooth hammerheads exhibit a greater reported range, extending further into cooler waters, than scalloped hammerheads (Compagno et al. 2005; De Bruyn et al. 2005; Van der Elst 1981). This may contribute to the different regional distribution patterns seen in the samples. In parallel, cooler waters require a greater degree of fluidity of lipids to provide greater buoyancy (Ballantyne 1997), which may underlie the increased levels of PUFA, especially n-3PUFA, seen in the smooth hammerhead livers. This may also reflect adaptation of lipid profiles to increase fluidity to counteract the reduction caused by the temperature decrease. At the same time, this may also reflect a greater dietary intake and/or a greater availability within the foodweb. This could also explain the reduction in liver PUFA with the transition from Spring to Summer as water temperatures increase. Minor dietary composition changes are unlikely to be the reason, as both species are reported to predate on a similar profile of prey (Cliff and Wilson 1986; Compagno et al. 2005). However, changes in the amount predated, rather than prey species, might explain the differences seen between the two hammerhead species in similar geographical regions. Overall, little work has been published on the diet of either hammerhead species, although Bush (2004) reported on limited variability of the diet in juvenile scalloped hammerheads around Hawaii.

This study has shown the comparative spectra of total lipid and fatty acids in two species of elasmobranch commonly found off the Indian Ocean coast of South Africa. The results are in close accordance with earlier publications on other shark species from the same region (Davidson and Cliff 2003, 2011; Davidson et al. 2011a, [b]).

## Abbreviations

KZN: KwaZulu-Natal
KZNSB: KwaZulu-Natal Sharks Board
NSB: Natal Sharks Board.
